# PLGA Nanoparticles Containing VCAM-1 Inhibitor Succinobucol and Chemotherapeutic Doxorubicin as Therapy against Primary Tumors and Their Lung Metastases

**DOI:** 10.3390/pharmaceutics15020349

**Published:** 2023-01-20

**Authors:** Jie Wang, Fengling Wang, Dandan Xie, Min Zhou, Jiaxing Liao, Hongliang Wu, Yue Dai, Jingbin Huang, Yu Zhao

**Affiliations:** 1 Department of Pharmacy, University-Town Hospital of Chongqing Medical University, Chongqing 401331, China; 2 Department of Pharmacy, The Second Affiliated Hospital of Army Medical University, Chongqing 400037, China

**Keywords:** co-delivery, PLGA nanoparticles, anti-metastasis, anti-tumor, VCAM-1 inhibitor, chemotherapeutics

## Abstract

The treatment of malignant tumors is usually accompanied by poor prognosis due to metastasis of tumor cells. Hence, it is crucial to enhance anti-metastasis efficacy when anti-tumor treatments are conducted. It has been reported that the vascular cell adhesion molecule-1 (VCAM-1) is highly expressed on the surface of tumor cells and plays an essential role in the metastasis of tumor cells. Thus, reducing VCAM-1 expression offers hope for inhibiting the metastasis of tumor cells. Evidence has shown that succinobucol (Suc) can selectively and efficiently inhibit VCAM-1 expression. Inspired by these, we designed dual drug-loaded PLGA nanoparticles (Co-NPs) to co-deliver VCAM-1 inhibitor Suc and the chemotherapeutic doxorubicin (Dox) which could both effectively suppress primary melanoma and its lung metastases. Co-NPs were composed of PLGA encapsulated Suc and Dox as hydrophobic cores and DSPE-mPEG_2000_ as surface modification materials. With an appropriate particle size (122.4 nm) and a negatively charged surface (−6.77 mV) we could achieve prolonged blood circulation. The in vitro experiments showed that Co-NPs had potent cytotoxicity against B16F10 cells and could significantly inhibit VCAM-1 expression and migration of B16F10 cells. Additionally, the in vivo experiments showed that Co-NPs could efficiently suppress not only primary melanoma but also its lung metastases. In conclusion, PLGA nanoparticles containing VCAM-1 inhibitor Suc and chemotherapeutic Dox as therapy against primary tumors and their lung metastases provides a promising drug delivery strategy for the treatment of metastatic malignant tumors.

## 1. Introduction

Malignant tumors remain the main cause of death globally, seriously threatening the health of humanity [1]. Additionally, the treatment of malignant tumors is still a major challenge for the medical community and usually accompanied by poor prognosis due to the metastasis of tumor cells [2,3,4]. Therefore, it is crucial to enhance the anti-metastasis effects at the same time anti-primary tumor treatments are conducted.

The metastasis of tumor cells is a highly complex multi-stage process that includes tumor cells invading the surrounding tissue from the primary tumor site, infiltrating the blood circulation, and the circulating tumor cells extravagating into distal tissues, finally forming metastases [5]. The latest research on anti-metastasis drugs mainly focused on decreasing the adhesion of tumor cells, inhibiting the degradation of proteolytic enzymes to the basement membrane, and the formation of tumor neovascularization [6,7,8]. Although several drugs had entered into clinical trials (TNP470, thalidomide, etc.), many of them did not achieve the expected purposes [9,10,11,12]. Thus, the development of new anti-metastasis drugs is extraordinarily crucial for the treatment of metastatic malignant tumors.

Vascular cell adhesion molecule-1 (VCAM-1) is a cytokine-induced adhesion molecule which is highly expressed on the surface of tumor cells [13]. Researchers detected that VCAM-1 plays a vital role in the metastasis of tumor cells and can be a potential therapeutic target in tumor metastasis [14,15,16,17,18]. Therefore, inhibiting VCAM-1 expression has attracted much attention in the treatment of metastatic malignant tumors. Succinobucol (Suc) is a probucol derivative with anti-inflammatory and anti-platelet aggregation effects [14,19]. It was reported that Suc could selectively and efficiently inhibit VCAM-1 expression, which indicates that Suc could be a promising drug for inhibiting the metastasis of tumor cells [20,21,22,23,24,25]. Cao et al. used Suc to self-assemble into nanoparticles with the triblock polymer poloxamer 188 to produce effects of anti-lung metastasis of breast cancer, which further confirmed the potential of Suc in the treatment of metastatic malignant tumors [23]. However, Suc has no inhibitory effect on the proliferation of tumor cells and is insoluble in water, which extremely limits its application in the treatment of metastatic malignant tumors. Therefore, combining Suc with chemotherapeutics may produce anti-metastasis and anti-tumor efficacy simultaneously, which could provide a promising strategy for the treatment of metastatic malignant tumors.

Doxorubicin (Dox), as a common broad-spectrum anti-tumor drug, can embed into the DNA to inhibit the synthesis of nucleic acids and is effective against multiple malignant tumors. However, the cardiotoxicity of Dox limits its application and evidence has shown that Dox can promote the metastasis of tumor cells [26,27,28,29,30,31]. Nechaeva et al. developed polymeric nanocarriers based on amphiphilic copolymers of N-vinyl-2-pyrrolidone and acrylic acid or allyl glycidyl ether, which could co-deliver paclitaxel and doxorubicin to achieve increasing bioavailability and decreasing cardiotoxicity [32,33]. Lu et al. developed doxorubicin-loaded micellar low-molecular-weight-heparin–astaxanthin nanoparticles which could inhibit breast cancer and its liver and lung metastases simultaneously [34]. Jiang et al. developed Dox-loaded liposomes decorated with a tumor-homing peptide, CREKA, which could serve as a valid therapeutic candidate for metastatic breast cancer [35]. Thus, designing a nano-system to co-deliver the VCAM-1 inhibitor Suc and the chemotherapeutic Dox might simultaneously eliminate primary tumors and their metastases, which will be greatly beneficial for the treatment of metastatic malignant tumors.

**Scheme 1:**
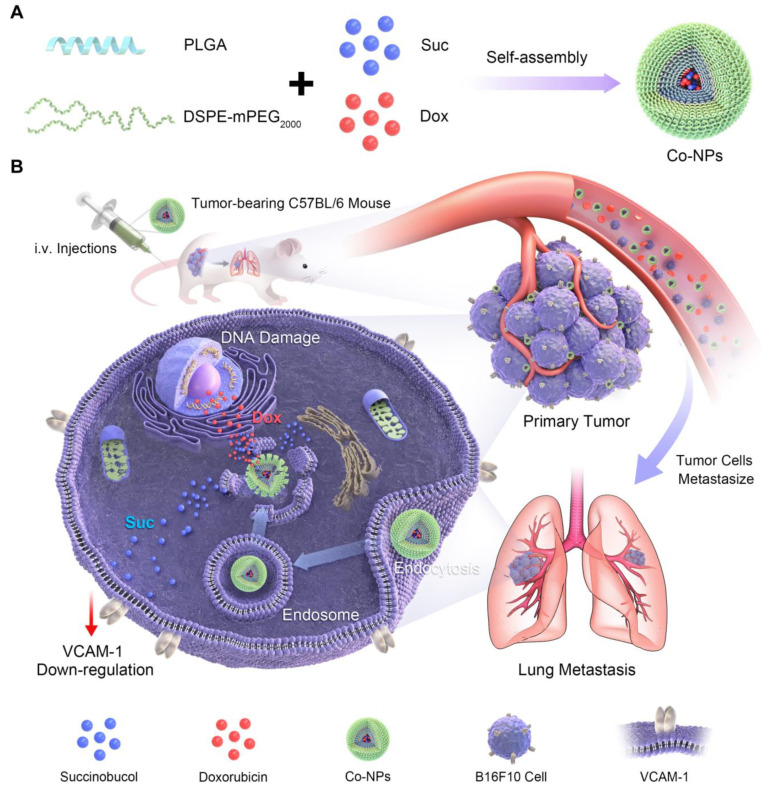
Schematic illustration. (**A**) Preparation of Co-NPs encapsulating Suc and Dox. (**B**) In vivo application of Co-NPs exerting effects of anti-primary tumor and anti-lung metastasis activity in metastatic melanoma. (Made using Blender 3.1 and Adobe Photoshop 21.2.3 software).

Herein, we developed dual drug-loaded nanoparticles (Co-NPs) to co-deliver the VCAM-1 inhibitor Suc and the chemotherapeutic Dox as therapy against primary tumors and their lung metastases. PLGA was used as a drug carrier due to its advantages of non-immunogenicity, non-toxicity, biocompatibility, and versatility [36]. Therefore, PLGA-encapsulated Suc and Dox as hydrophobic cores, and DSPE-mPEG_2000_ as surface modification material were used to form Co-NPs (Scheme 1) [37]. Our in vitro experiments confirmed that the prepared Co-NPs had favorable stability, hemocompatibility, strong cellular uptake, and potent cytotoxicity. In addition, Co-NPs exerted powerful inhibitory effects on cell migration and VCAM-1 expression due to Suc as a potent and selective VCAM-1 inhibitor. Furthermore, the efficacy of Co-NPs as anti-primary tumor and anti-lung metastasis agents was verified in primary melanoma and in melanoma lung metastasis tumor-bearing mouse models, and Co-NPs showed favorable safety in vivo. This work combines the VCAM-1 inhibitor Suc and the chemotherapeutic Dox in a well-designed nano-delivery system to suppress primary tumors and their lung metastases simultaneously, which provides a promising drug delivery strategy for the treatment of metastatic malignant tumors.

## 2. Materials and Methods

### 2.1. Materials

Doxorubicin was purchased from Meilunbio (Dalian, China). Succinobucol and VCAM-1 antibody were purchased from Santa Cruz Biotechnology (Dallas, TX, USA). Poly (lactic-co-glycolic acid) (PLGA, 50:50, 12,000 Da) was purchased from LACTEL Absorbable Polymers (Birmingham, AL, USA). DSPE-mPEG_2000_ was purchased from Peng Sheng Biotechnology (Shanghai, China). Acetonitrile was purchased from Honeywell (Charlotte, NC, USA). Phosphate buffer saline (PBS), 4, 6-diamidino-2-phenylindole (DAPI), 4% paraformaldehyde, FITC-labeled goat anti-mouse IgG (H + L), and Hoechst 33342 were purchased from Beyotime Biotechnology (Shanghai, China). Thiazolyl blue tetrazolium (MTT) was purchased from Sigma-Aldrich (St. Louis, MO, USA). APC-Annexin-V was purchased from BD Biosciences Pharmingen (San Diego, CA, USA). All other solvents and reagents were analytical.

### 2.2. B16F10 Cell Culture Methods

Mouse melanoma B16F10 cells were acquired from the American Type Culture Collection (ATCC, Manassas, VA, USA) and cultured in Dulbecco’s modified eagle medium (DMEM, Gibco, Waltham, MA, USA) supplemented with 10% heat-inactivated fetal bovine serum (FBS, Lonsera, Shanghai, China) and 1% penicillin–streptomycin (Beyotime, Shanghai, China). The cells were cultured in a cell culture incubator at 37 °C with 5% carbon dioxide.

### 2.3. Preparation and Characterization of Nanoparticles

Nanoparticles (NPs) were prepared using a self-assembly nano-precipitation method. Concisely, PLGA (3.0 mg), DSPE-mPEG_2000_ (1.5 mg), Dox (1.5 mg, free base), or/and Suc (1.5 mg) were all dissolved in 130 μL of dimethyl sulfoxide (DMSO). Additionally, soybean phospholipid (1.3 mg) was dissolved in 20 μL of methanol. Subsequently, this 150 μL of organic phase mixture was dropwise added to 3 mL of rapidly stirred (600 rpm) deionized water at room temperature. Eventually, the residual DMSO and methanol were removed using dialysis in deionized water to obtain Dox NPs, Suc NPs, and Co-NPs.

The size, polydispersity index (PDI), and zeta potential of all nanoparticles were detected using a dynamic light scattering (DLS) instrument (Zetasizer Nano ZS90, Malvern, UK). To observe the morphology of nanoparticles, Dox NPs, Suc NPs, and Co-NPs with a suitable concentration were dropped on copper nets covered with carbon support films (Zhongjingkeyi Technology, Beijing, China) and air dried overnight. Subsequently, samples were characterized using transmission electron microscopy (TEM, HT7700, Hitachi, Japan). To further assess the stability of Dox NPs, Suc NPs, and Co-NPs, nanoparticles were dissolved in PBS. The size, PDI, and zeta potential of all nanoparticles were consecutively detected using DLS at set time points (0 h, 2 h, 4 h, 6 h, 8 h, 12 h, 16 h, 24 h, 48 h).

To determine the drug loading efficiency (LE%) and the drug encapsulation efficiency (EE%) of Dox NPs, Suc NPs, and Co-NPs, the supernatants and subnatants of different nanoparticles were collected. Additionally, loaded and unloaded Dox and Suc were detected using high performance liquid chromatography (HPLC, Agilent 1200, Santa Clara, CA, USA) through a reverse-phase column (Phenomenex Luna 5u C18 100 A, 250 × 4.6 mm, 5 μm). For Dox, the mobile phase was composed of acetonitrile and water adjusted with H_3_PO_4_ to pH 3.0 (28/72, *v/v*), the flow rate was 1 mL/min, the temperature of the column was 30 °C, the detection wavelength was 254 nm, and the injection volume was 10 μL. For Suc, the mobile phase was composed of acetonitrile and water with 0.1% trifluoroacetic acid (98/2, *v/v*), the flow rate was 1.5 mL/min, the temperature of column was 30 °C, the detection wavelength was 242 nm, and the injection volume was 10 μL. Finally, the LE % and EE % of Dox and Suc for different nanoparticles were calculated as shown below:

$$\mathrm{LE}\;\%=\frac{\mathrm{Amount}\;\mathrm{of}\;\mathrm{drug}\;\mathrm{encapsulated}}{\mathrm{Amount}\;\mathrm{of}\;\mathrm{drug}\;\mathrm{encapsulated}+\mathrm{Amount}\;\mathrm{of}\;\mathrm{nanoparticles}}\times 100\%\;\mathrm{EE}\;\%=\frac{1-\mathrm{Amount}\;\mathrm{of}\;\mathrm{drug}\;\mathrm{unencapsulated}}{\mathrm{Amount}\;\mathrm{of}\;\mathrm{drug}\;\mathrm{used}}\times 100\%$$

### 2.4. Hemolysis Assay

To investigate the hemocompatibility of different nanoparticles, whole blood was collected from SD rats and centrifuged at 800 g/min for 5 min to obtain red blood cells (RBCs). Subsequently, the obtained RBCs were diluted to 10% with PBS and 0.3 mL of diluted RBC suspension was added into 1.5 mL centrifuge tubes, then centrifugated at 800 g/min for 5 min. Next, Dox NP, Suc NP, and Co-NP solutions with different concentrations ranging from 25 to 400 μg/mL were added into the RBCs suspension with water as positive control and PBS as negative control, then incubated on a horizontal shaker (KS3000i, IKA, Stauffen, Germany) at 100 rpm, at 37 °C for 3 h. Eventually, the absorbance of the supernatant of each tube at 541 nm was measured with a microplate reader (Multiskan GO 1510, Thermo Fisher, Waltham, MA, USA) [38]. The hemolysis rates of different nanoparticles were calculated as shown below:

$$\mathrm{Hemolysis}\;\%=\frac{\left({\mathrm{OD}}_{\mathrm{sample}}-{\mathrm{OD}}_{\left(-\right)\mathrm{control}}\right)}{{\mathrm{OD}}_{\left(+\right)\mathrm{control}}-{\mathrm{OD}}_{\left(-\right)\mathrm{control}}}\times 100\%$$

OD represents the optical density.

### 2.5. In Vitro Drug Release

HPLC was employed to investigate the drug release behavior of Dox NPs, Suc NPs, and Co-NPs and the conditions for chromatographic measurements of Dox and Suc were similar to those in the previous chapter. Different nanoparticles were prepared according to the method described above, and each group was placed in a dialysis membrane bag (MWCO = 3500). Subsequently, the dialysis membrane bags were placed in 30 mL of PBS with 0.1% Tween-80 at pH 5.0 or 7.4, then maintained on a horizontal shaker (KS3000i, IKA, Stauffen, Germany) at 100 rpm, at 37 °C without light. 3 mL of PBS was removed at set time points (0.5 h, 1 h, 2 h, 4 h, 6 h, 8 h, 20 h, 24 h, 36 h, 48 h, 60 h, 72 h) and 3 mL of fresh PBS was supplemented. Eventually, the cumulative release of Dox and Suc was quantified using HPLC.

### 2.6. In Vitro Cellular Uptake

An inverted fluorescence microscope (IFM) and flow cytometry (FCM) were used to qualitatively and quantitatively analyze the cellular uptake of Co-NPs by B16F10 cells. For qualitative analysis [39,40,41], 1 × 10^5^ B16F10 cells per well were seeded into 6-well plates and then cultured for 24 h at 37 °C. Subsequently, the cells were treated with free Dox (20 μg/mL) and Co-NPs (20 μg/mL Dox equivalents) at 37 °C for 1 h and 2 h. After incubation, the cells were washed with PBS and fixed with 4% paraformaldehyde. Subsequently, cell nuclei were stained with DAPI (5 mg/mL). Eventually, imaging of cell fluorescence was performed using an IFM (DMi8, Leica, Wetzlar, Germany). For quantitative analysis, as in the previous steps, the cells were trypsinized, centrifuged, and re-suspended in cold PBS after washing with PBS to detect the intensity of cell fluorescence using FCM.

### 2.7. In Vitro Cytotoxicity Assay

An MTT assay was employed to evaluate the in vitro cytotoxic effect on B16F10 cells treated with different drug formulations. Concisely, 5 × 10^3^ B16F10 cells per well were seeded into 96-well plates and then cultured for 24 h at 37 °C. After the treatments with different concentrations of Dox, Suc, Dox + Suc, Dox NPs, Suc NPs, and Co-NPs for 24 or 48 h at 37 °C, the cells were incubated with MTT (5 mg/mL, 20 μL per well) for another 4 h. Eventually, 150 μL of DMSO per well was used to dissolve the formazan of MTT before measurements using a microplate reader (Multiskan GO 1510, Thermo Fisher, Waltham, MA, USA) at 570 nm.

### 2.8. In Vitro Apoptosis Assay

FCM was employed to analyze in vitro apoptosis of B16F10 cells induced by different drug formulations. Briefly, 1 × 10^5^ B16F10 cells per well were seeded into 6-well plates and then cultured for 24 h at 37 °C. After the treatments with Dox, Suc, Dox + Suc, Dox NPs, Suc NPs, and Co-NPs (1.0 μg/mL Dox equivalents and 1.6 μg/mL Suc equivalents) for 24 h at 37 °C, the cells were subjected to incubation with Annexin V-APC/DAPI for 15 min. Eventually, the intensity of fluorescence was measured using FCM.

### 2.9. In Vitro Inhibition of Cell Migration

A wound-healing assay and a transwell migration assay were employed to investigate the in vitro anti-metastatic efficacy of different drug formulations.

For the wound-healing assay, 1 × 10^5^ B16F10 cells per well were seeded into 6-well plates and then cultured for 24 h at 37 °C. After forming a well-demarcated gap, cells were washed with PBS three times. Subsequently, Dox, Suc, Dox + Suc, Dox NPs, Suc NPs, and Co-NPs (1.0 μg/mL Dox equivalents and 1.6 μg/mL Suc equivalents) were added to each well and cultured for further 24 h at 37 °C. Eventually, the level of wound healing was observed on an optical microscope (DP27, Olympus, Tokyo, Japan) through obtaining images at 0 h and 24 h.

For the transwell migration assay [42], 200 μL of serum-free DMEM containing 2.5 × 10^4^ B16F10 cells and Dox, Suc, Dox + Suc, Dox NPs, Suc NPs, and Co-NPs (1.0 μg/mL Dox equivalents and 1.6 μg/mL Suc equivalents) per well was added into the upper section of chambers. Subsequently, 600 μL of DMEM supplemented with 30% FBS and containing Dox, Suc, Dox + Suc, Dox NPs, Suc NPs, and Co-NPs (1.0 μg/mL Dox equivalents and 1.6 μg/mL Suc equivalents) per well was added into the lower section of chambers. After incubation for 24 h at 37 °C, the non-migrated cells on the surface of the upper section of each chamber were cautiously removed and the migrated cells on the surface of the lower section of each chamber were fixed with 4% paraformaldehyde and stained with 0.1% crystal violet solution. Eventually, the level of cell migration was observed using an optical microscope (DMi8, Leica, Wetzlar, Germany), and the migratory cells per field were quantified using ImageJ software.

### 2.10. Immunofluorescence Assay of VCAM-1 Expression

The immunofluorescence assay was employed to determine the inhibitory effect of different drug formulations on VCAM-1 expression of B16F10 cells [23,25]. Concisely, 2.5 × 10^4^ B16F10 cells per well were seeded into 24-well plates with round glass coverslips (ø 10 mm) and then cultured for 24 h at 37 °C. After treatment with Dox, Suc, Dox + Suc, Dox NPs, Suc NPs, and Co-NPs (1.0 μg/mL equivalents of Dox and 1.6 μg/mL equivalents of Suc) for 24 h at 37 °C, cells were fixed with 4% paraformaldehyde and then incubated with the primary antibody against VCAM-1 and FITC-labeled goat anti-mouse IgG (H + L) in accordance with the manufacturer’s protocols. The nuclei were stained with Hoechst 33342. Eventually, the immunofluorescence of cells was imaged using a confocal laser scanning microscope (CLSM, LSM800, Zeiss, Jena, Germany).

### 2.11. Animal Studies

Female C57BL/6 mice (8 weeks) and female Sprague-Dawley (SD) rats (8 weeks) were acquired from the Laboratory Animal Center of Army Medical University. All experiments involving animals were approved by the Laboratory Animal Welfare and Ethics Committee of Army Medical University (AMUWEC2022454).

### 2.12. In Vivo Anti-Primary Tumor Efficacy

B16F10 melanoma tumor-bearing female C57BL/6 mice were used to evaluate the in vivo anti-primary tumor efficacy of different drug formulations. A total of 1 × 10^6^ B16F10 cells per mouse were subcutaneously injected to generate B16F10 melanoma models. Four days after inoculation, the mice were randomly divided into seven groups (eight mice per group) and saline, Dox, Suc, Dox + Suc, Dox NPs, Suc NPs, and Co-NPs (5.0 mg/kg Dox equivalents, 8.0 mg/kg Suc equivalents) were administrated via the tail vein on 3 consecutive days. Meanwhile, the tumor volume and the body weight of mice were monitored using vernier calipers and an electronic balance every two days (V=(L×W^2)/2, *V* represents the length of tumor, *W* represents the width of tumor). All mice were sacrificed on day 12 and autopsied to obtain the blood, tumor tissue, and major organs.

The obtained tumor tissues and major organs, including the heart, liver, spleen, lung, and kidney, were fixed in 4% paraformaldehyde for several days and then embedded in paraffin blocks. Subsequently, each block was cut into 5 μm thick sections which were stained with hematoxylin and eosin (H&E), and TUNEL. The H&E- and TUNEL-stained pathological sections were visualized with an optical microscope (DMi8, Leica, Wetzlar, Germany). Furthermore, the VCAM-1 expression on tumor tissues was determined using an immunofluorescence assay as in the previous chapter. In addition, the serum separated from the blood was used to investigate the level of main biochemical factors (AST, ALT, CREA, UREA, CK, LDH).

### 2.13. In Vivo Anti-Lung Metastasis Efficacy

B16F10 melanoma lung metastasis-bearing female C57BL/6 mice were used to evaluate the in vivo anti-lung metastasis efficacy of different drug formulations. A total of 5 × 10^5^ B16F10 cells per mouse were injected via the tail vein to generate B16F10 melanoma lung metastatic models. Ten days after inoculation, the mice were randomly divided into seven groups (eight mice per group) and saline, Dox, Suc, Dox + Suc, Dox NPs, Suc NPs, and Co-NPs (5.0 mg/kg Dox equivalents, 8.0 mg/kg Suc equivalents) were administrated via the tail vein on 4 consecutive days. Meanwhile, the body weight of mice was monitored every two days. All mice were sacrificed on day 21, and the lungs of mice were cautiously taken away. Subsequently, the lungs were photographed, and the metastatic nodules on the lung surface were counted. Eventually, H&E staining, TUNEL staining, and immunofluorescence assays were employed to evaluate the morphological changes, the level of apoptosis, and the VCAM-1 expression on tumor tissues as in the previous chapter.

### 2.14. Statistical Analysis

Data are presented as mean ± standard deviation (SD). Statistical analyses were conducted using unpaired two-tailed Student’s *t*-tests. Statistical significance was defined as * *p* < 0.05, ** *p* < 0.01, *** *p* < 0.001, and **** *p* < 0.0001.

## 3. Results

### 3.1. Characterization

**Figure 1:**
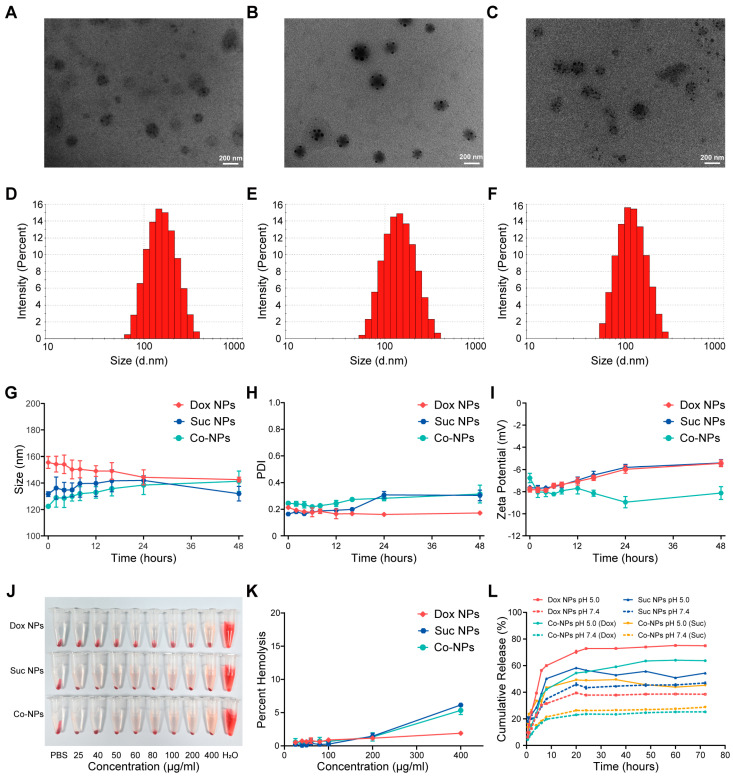
Characterization of different nanoparticles. (**A**–**C**) TEM images of Dox NPs (**A**), Suc NPs (**B**), and Co-NPs (**C**). (**D**–**F**) Particle size distribution of Dox NPs (**D**), Suc NPs (**E**), and Co-NPs (**F**). (**G**–**I**) Size (**G**), PDI (**H**), and zeta potential (**I**) of Dox NPs, Suc NPs, and Co-NPs in PBS (pH 7.4) within 48 h (*n* = 3). (**J**) Hemolysis assay of Dox NPs, Suc NPs, and Co-NPs at different concentrations ranging from 25 to 400 μg/mL. (**K**) Quantitative characterization of hemolysis (*n* = 3). (**L**) Drug release behavior profiles of Dox NPs, Suc NPs, and Co-NPs in PBS at pH 5.0 and 7.4 (*n* = 3).

The different drug-loaded nanoparticles composed of drugs, PLGA, and DSPE-mPEG_2000_ were successfully self-assembled through the nanoprecipitation method. Firstly, TEM images of different nanoparticles showed that these nanoparticles all had spherical profiles (Figure 1A–C). Meanwhile, the particle size of different nanoparticles was measured using DLS. As shown in Figure 1D–F, Dox NPs, Suc NPs, and Co-NPs all had the desired particle size with a diameter ranging from 122 to 156 nm and a narrow size distribution with a PDI from 0.164 to 0.245. Additionally, as shown in Figure S1, all nanoparticles showed good nanometer characteristics and dispersion. As shown in Table 1, for Co-NPs, the drug loading efficiency of Dox and Suc was 4.02% and 6.54%, respectively; the drug encapsulation efficiency of Dox and Suc was 84.30% and 94.29%, respectively. In addition, the results of stability experiment showed that none of these nanoparticles exhibited significant changes in particle size, PDI, and zeta potential in PBS within 48 h of incubation (Figure 1G–I). It was also confirmed that both particle size and PDI of all nanoparticles exhibited no significant changes in PBS within 7 days (Figure S2A,B), further suggesting their favorable storage stability.

Great compatibility of nanoparticles with RBCs is a critical factor for in vivo application. Therefore, a hemolysis assay of nanoparticles was performed. As shown in Figure 1J,K, there was no significant hemolysis for different nanoparticle formulations even at a high concentration of 400 μg/mL, and the hemolysis rates of all nanoparticles were less than 6.15%, suggesting that all nanoparticles showed favorable hemocompatibility and safety for intravenous injection.

### 3.2. In Vitro Drug Release

The drug release behavior of different nanoparticles at pH 5.0 and 7.4 was measured using HPLC, and the results are presented in Figure 1L. Both Dox and Suc exhibited biphasic release patterns that included a burst release at the beginning followed by a sustained release. Notably, when Dox and Suc were co-loaded in nanoparticles, the cumulative release of Dox and Suc at pH 5.0 within 72 h had a slight decrease to 63.68% and 45.21%, respectively, compared with Dox NPs (74.94%) and Suc NPs (54.35%). It could easily be detected that both Dox and Suc were released faster and more at pH 5.0 than at pH 7.4, indicating consistent results with previous data that nanoparticles could maintain stability at pH 7.4. Thus, nanoparticles could have more prolonged circulation time in the blood and release drugs faster in a mildly acidic environment, which benefits their anti-tumor and anti-metastasis efficacy.

### 3.3. In Vitro Cellular Uptake

**Figure 2:**
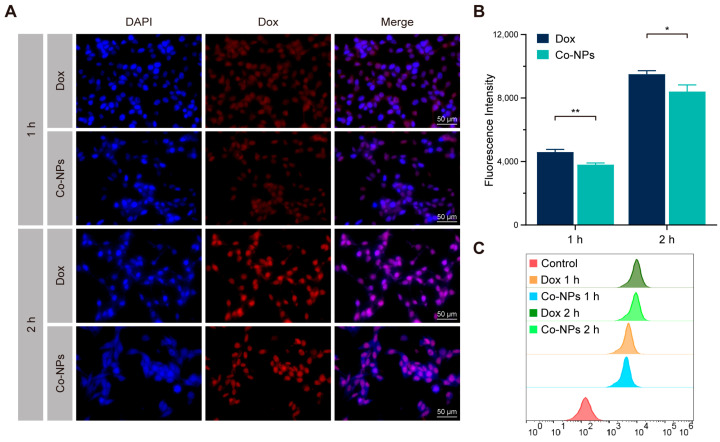
In vitro cellular uptake. (**A**) Cellular uptake of free Dox and Co-NPs after incubation with B16F10 cells for 1 h and 2 h. Scale bar = 50 μm. (**B**,**C**) Quantitative analysis of cellular uptake of free Dox and Co-NPs by B16F10 cells using FCM (*n* = 3). (* *p* < 0.05, ** *p* < 0.01).

A rapid and massive cellular uptake is considered to play an essential role in anti-tumor activity and subsequent cytotoxicity. Therefore, the cellular internalization of Co-NPs (an equivalent concentration for 20 μg/mL of Dox) was evaluated on B16F10 cells and the results are shown in Figure 2. The Dox groups only showed slightly stronger fluorescence intensity in contrast with the Co-NPs groups regardless of incubation for 1 h or 2 h (Figure 2A). This observation is quite acceptable as Dox enters cells mainly through the way of passive diffusion, while nanoparticles enter cells mainly through active pathways such as endocytosis [43,44,45]. All groups displayed a time-dependent cellular uptake as the cellular uptake within 2 h was significantly higher than that within 1 h. The qualitative analysis using FCM showed a similar tendency (Figure 2B,C).

### 3.4. In Vitro Cytotoxicity and Apoptosis

**Figure 3:**
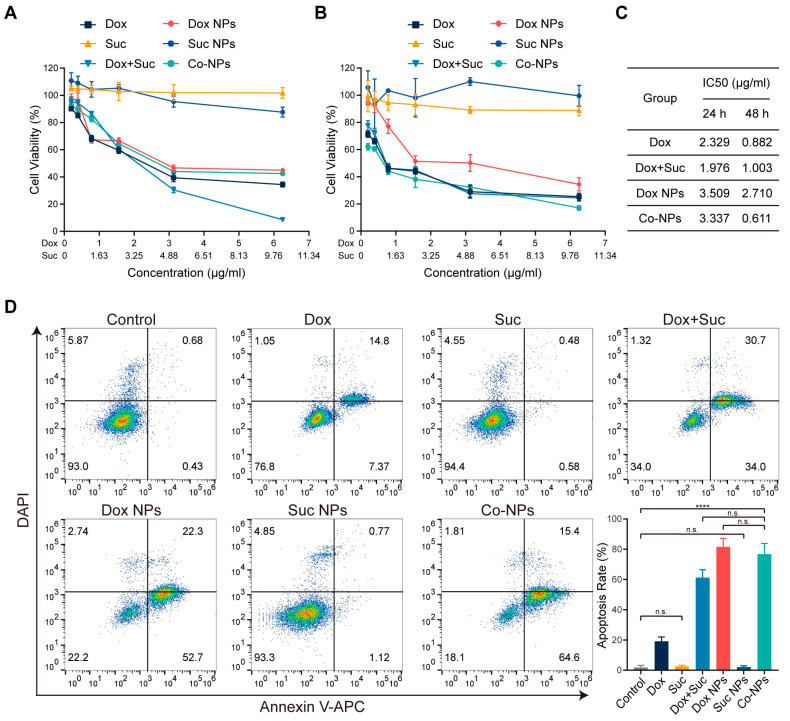
In vitro cytotoxicity and apoptosis. (**A**,**B**) In vitro cytotoxicity of Dox, Suc, Dox + Suc, DOX NPs, Suc NPs, and Co-NPs against B16F10 cells within 24 h (**A**) and 48 h (**B**), respectively (*n* = 3). (**C**) IC50 values of different free drugs and nanoparticles within 24 and 48 h. (**D**) In vitro apoptosis of B16F10 cells induced by Dox, Suc, Dox + Suc, Dox NPs, Suc NPs, and Co-NPs within 24 h (*n* = 3). (n.s., not significant, **** *p* < 0.0001).

Next, to investigate the effect of different drug formulations on cell proliferation of B16F10 cells, MTT assays was conducted. As shown in Figure 3A,B, neither Suc nor Suc NPs showed evident inhibition of the proliferation of B16F10 cells at experimental concentrations, while the treatments including Dox (Dox, Dox + Suc, Dox NPs, and Co-NPs) caused a dose-dependent decrease in cell viability within 24 and 48 h. The IC50 values of different free drugs and nanoparticles are shown in Figure 3C. The Dox and Suc combined treatments (IC50 of Dox + Suc: 1.976 μg/mL, IC50 of Co-NPs: 3.337 μg/mL) showed higher cytotoxicity within 24 h compared with cytotoxicity of Dox (IC50 of Dox: 2.329 μg/mL, IC50 of Dox NPs: 3.509 μg/mL) treatments alone, demonstrating that there was a synergistic cytotoxicity of Dox and Suc. After loading drugs to nanoparticles, the cytotoxicity decreased within 24 h as the endocytosis pathway of the drugs changed. On the contrary, Co-NPs (IC50: 0.611 μg/mL) showed the highest inhibition of the proliferation of B16F10 cells within 48 h, which might be due to the controlled release from nanoparticles and the synergistic cytotoxicity of Dox and Suc [46,47].

Annexin V-APC/DAPI double staining was employed to investigate the apoptosis induced by different drug formulations (Figure 3D). Consistent with the results of the cytotoxicity experiments, neither Suc nor Suc NPs induced apparent apoptosis of B16F10 cells, which further confirmed the non-toxic characteristics of Suc at experimental concentrations against B16F10 cells. Contrariwise, Dox induced apoptosis in 19.06 ± 2.93% of B16F10 cells. However, a noticeable increase in apoptosis was observed in the Dox + Suc (61.12 ± 5.36%) and Co-NP (76.67 ± 7.11%) groups, which further certified the synergistic cytotoxicity of Dox and Suc.

### 3.5. In Vitro Anti-Metastasis Effects

**Figure 4:**
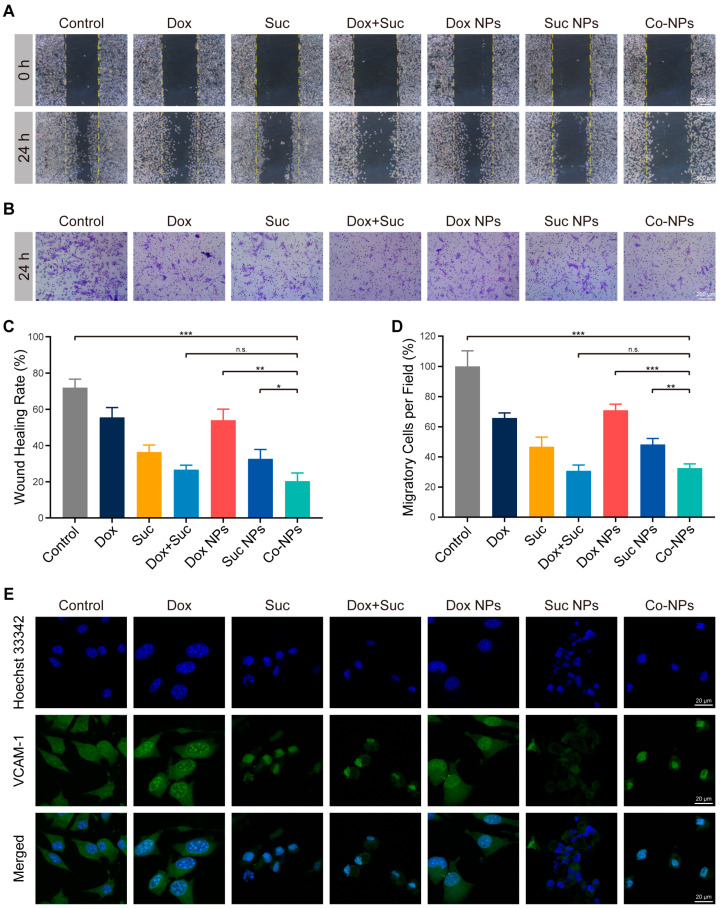
In vitro anti-metastasis effects and inhibitory effects on VCAM-1 expression. (**A**) Typical images of the wound healing assay of different free drugs and nanoparticles. Scale bars = 500 μm. (**B**) Typical images of the transwell migration assay of different free drugs and nanoparticles. Scale bar = 200 μm. (**C**) Quantitative analysis of the wound healing assay (*n* = 3). (**D**) Quantitative analysis of the transwell migration assay (*n* = 3). (**E**) Immunofluorescence assay of VCAM-1 expression on B16F10 cells after treatment with different free drugs and nanoparticles. Scale bars = 20 μm. (n.s., not significant, * *p* < 0.05, ** *p* < 0.01, *** *p* < 0.001).

Wound healing assays and the transwell migration assays were employed to verify the inhibitory effects of different drug formulations on cell migration. As shown in Figure 4A, the control group showed an apparent trend of increasing number of cells migrating to the center of the scratched gap after 24 h of incubation. By contrast, the migratory ability of B16F10 cells was evidently inhibited after the treatments including Suc. The wound healing rates in the Suc- and Suc NP-treated groups decreased to 36.33 ± 3.91% and 32.58 ± 5.25%, respectively (Figure 4C). When Suc was combined with Dox, the wound healing rates in the Dox + Suc- and Co-NP-treated groups further decreased to 26.59 ± 2.55% and 20.28 ± 4.58%, respectively, indicating that Dox enhanced the inhibitory effect of Suc on cell migration. In addition, groups treated with Suc (Suc, Dox + Suc, Suc NPs, and Co-NPs) showed an obviously decreasing number of migrating B16F10 cells in the 30% FBS-induced transwell cell-migration assay (Figure 4B). The number of migrating cells in the Suc- and Suc NP-treated groups was 46.72 ± 6.42% and 48.15 ± 4.09% of the negative control, respectively, and further decreased to 30.74 ± 3.84% in the Dox + Suc-treated group and 32.58 ± 2.82% in the Co-NP-treated group (Figure 4D). The wound healing assay and the transwell migration assay both revealed that Suc could effectively inhibit the migration of B16F10 cells and Dox could enhance the inhibitory effect of Suc on cell migration.

### 3.6. Inhibitory Effects on VCAM-1 Expression

It has been reported that in the lung metastasis of tumor cells, the high expression of VCAM-1 on the surface of tumor cells plays a crucial role in the formation of lung metastasis colonies [14,15,48]. Hence, immunofluorescence assays were employed to evaluate the inhibitory effect of different drug formulations on VCAM-1 expression. As shown in Figure 4E, the control group showed strong green fluorescence, confirming the high VCAM-1 expression on B16F10 cells [49]. Similar results were observed in the Dox- and Dox NP-treated groups, which indicated that Dox had almost no inhibitory effects on VCAM-1 expression. Contrariwise, a noticeable reduction in VCAM-1 expression was observed after treatments including Suc (Suc, Dox + Suc, Suc NPs, Co-NPs). Notably, a more significant decrease in VCAM-1 expression was observed after treatment with Co-NPs, verifying that Co-NPs could effectively inhibit VCAM-1 expression on B16F10 cells.

### 3.7. In Vivo Anti-Primary Tumor Efficacy

**Figure 5:**
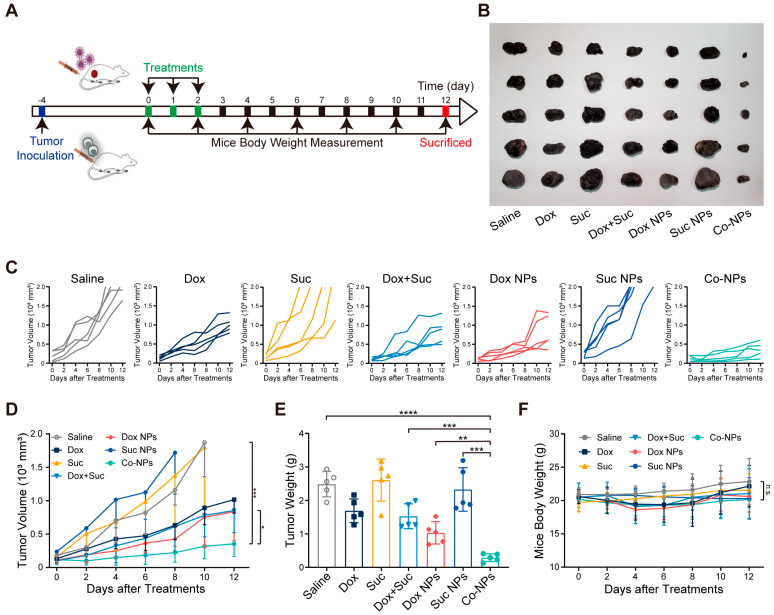
In vivo anti-primary tumor efficacy. (**A**) Workflow for the treatment of B16F10 melanoma tumor-bearing mice. On day 0 (defined as the 5th day after tumor inoculation), day 1, and day 2, mice were administrated with saline, Dox, Suc, Dox + Suc, Dox NPs, Suc NPs, and Co-NPs. Tumor volume and body weight of mice were monitored every 2 days from day 0 to day 12. (**B**) Photograph of tumors collected on day 12. (**C**) Time-dependent tumor growth curves of mice for individual groups during the treatments. (**D**) Time-dependent tumor growth curves of all groups during the treatments. (**E**) Tumor weight of mice for individual groups after different treatments. (**F**) Changes in body weight of mice during the treatment. (n.s., not significant, * *p* < 0.05, ** *p* < 0.01, *** *p* < 0.001, **** *p* < 0.0001).

To investigate the in vivo anti-primary tumor efficacy of different drug formulations, we first established a B16F10 melanoma-bearing C57BL/6 mouse model (Figure 5A). Figure 5B shows a photograph of harvested tumors from differently treated groups. As shown in Figure 5C,D, the curves of tumor volume growth showed that the tumors of the saline-, Suc- and Suc NP-treated groups grew quickly, while the tumor growth was apparently suppressed in the groups treated using formulations including Dox. In particular, Co-NPs demonstrated the most potent anti-tumor efficacy, which was better than that of two separate monotherapies. The results were consistent with the in vitro anti-tumor results, indicating that Dox and Suc had also synergistic anti-tumor efficacy in vivo. Furthermore, the tumor weight of differently treated groups showed an analogous tendency that Co-NPs could produce the most potent anti-tumor efficacy in B16F10 melanoma-bearing C57BL/6 mice (Figure 5E).

Meanwhile, to evaluate the safety of Co-NPs in vivo, we monitored the body weight of mice during the treatment. There were no significant changes in the body weight of mice in the Co-NP-treated group during the whole treatment, representing the favorable safety of Co-NPs in vivo (Figure 5F). In addition, no significant histological damage was confirmed in the Co-NP-treated group by H&E staining of the tissues of heart, lung, liver, spleen, and kidney (Figure S3A), further suggesting that Co-NPs had little toxicity to main organs. Moreover, the typical biochemical indicators of the function of liver, kidney, and heart were also assayed, including AST, ALT, CREA, UREA, CK, and LDH. As shown in Figure S3B–G, all indexes of the Co-NP-treated group were within the normal numerical ranges compared to the saline-treated group, further demonstrating that Co-NPs were safe in vivo.

**Figure 6:**
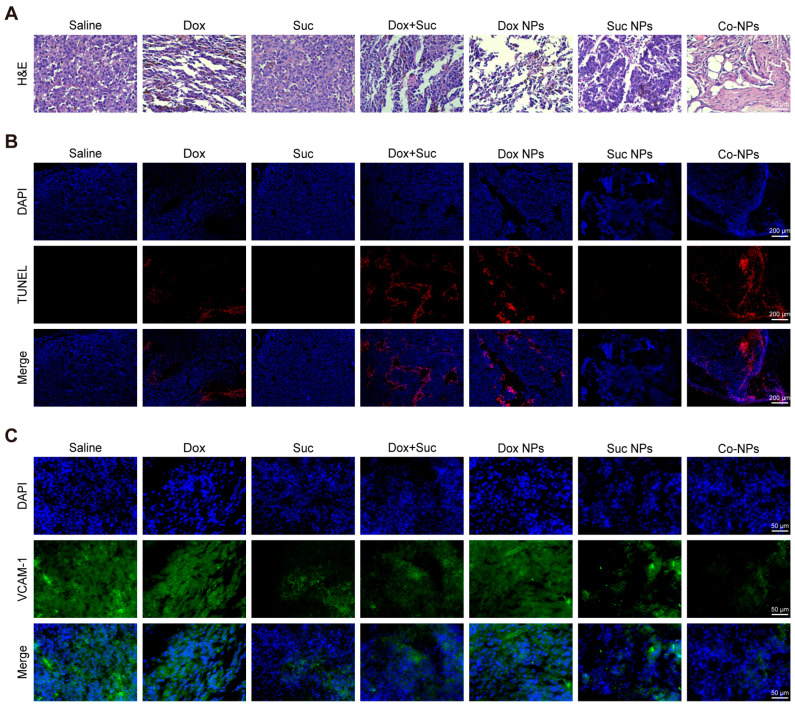
In vivo anti-primary tumor efficacy. (**A**) H&E staining of tumor tissues after different treatments. Scale bar = 50 μm. (**B**) TUNEL staining of tumor tissues after different treatments. Scale bars = 200 μm. (**C**) Immunofluorescence staining of VCAM-1 expression on tumor tissues after different treatments. Scale bars = 50 μm.

Moreover, the tumor tissues were stained with H&E and TUNEL to investigate the histological changes and apoptosis after different treatments. As shown in Figure 6A, compact nuclei of tumor cells could be easily observed in the tumor tissues of the saline-, Suc-, and Suc NP-treated groups, while fewer nuclei of tumor cells were observed in the treatments including Dox. The Co-NP-treated group exhibited the largest area of necrosis, demonstrating its remarkable inhibitory effect on tumor growth. In addition, the results of TUNEL staining revealed that Co-NPs induced the highest level of apoptosis among differently treated groups (Figure 6B), which further confirmed that Dox and Suc had synergistic anti-tumor efficacy.

Finally, immunofluorescence assays were performed to evaluate VCAM-1 expression on B16F10 cells of the tumor tissues after different treatments. As shown in Figure 6C, VCAM-1 expression in the Dox- and Dox NP-treated groups was close to the negative control, while VCAM-1 expression in the treatments including Suc was significantly reduced, confirming that Suc could effectively inhibit VCAM-1 expression. Notably, the Co-NP-treated group presented the lowest VCAM-1 expression, further certifying that Dox enhanced the inhibitory effects of Suc on VCAM-1 expression.

### 3.8. In Vivo Anti-Lung Metastasis Efficacy

**Figure 7:**
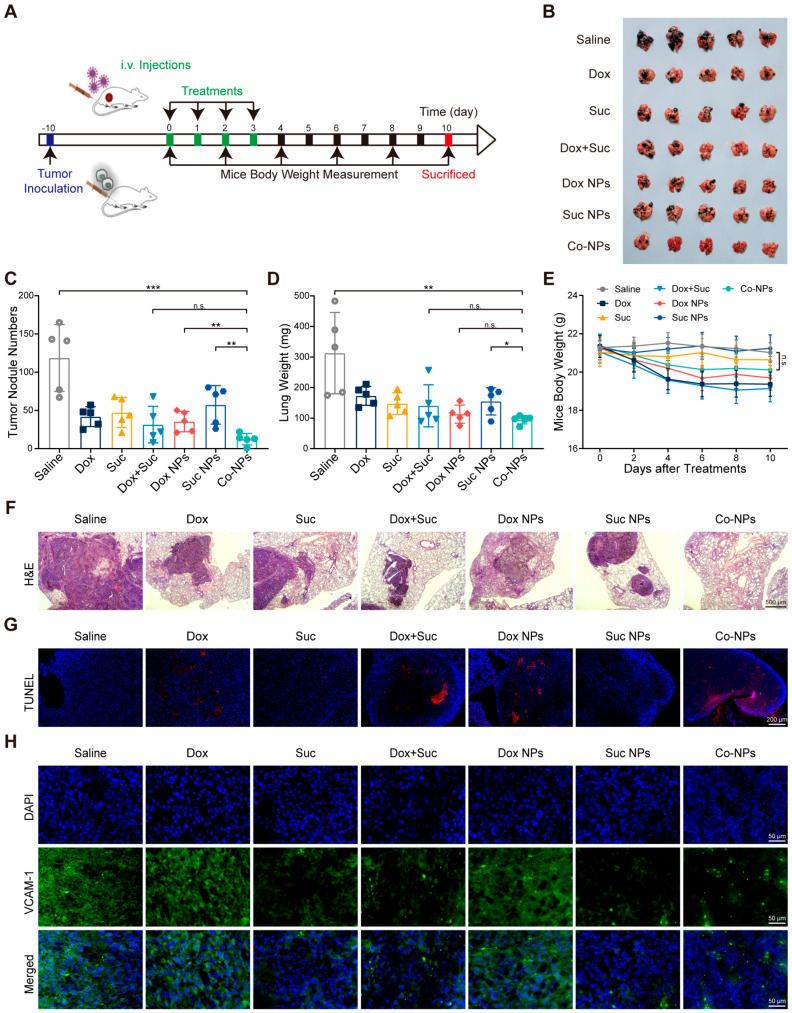
In vivo anti-lung metastasis efficacy. (**A**) Workflow for the treatment of B16F10 melanoma lung metastasis-bearing mice. On day 0 (defined as the 11th day after tumor inoculation), day 1, day 2, and day 3, mice were administrated with saline, Dox, Suc, Dox + Suc, Dox NPs, Suc NPs, and Co-NPs. Body weight of mice was monitored every 2 days from day 0 to day 10. (**B**) Photograph of lungs collected on day 11. (**C**) Tumor nodule numbers on the harvested lungs from individual groups after different treatments. (**D**) Lung weight of mice for individual groups after different treatments. (**E**) Changes of mice body weight during the treatments. (**F**) H&E staining of lung tissues after different treatments. Scale bar = 500 μm. (**G**) TUNEL staining of lung tissues after different treatments. Scale bar = 200 μm. (**H**) Immunofluorescence assay of VCAM-1 expression on lung tissues after different treatments. Scale bars = 50 μm. (n.s., not significant, * *p* < 0.05, ** *p* < 0.01, *** *p* < 0.001).

To further investigate the in vivo anti-lung metastasis efficacy of Co-NPs, we established a B16F10 melanoma lung metastasis-bearing C57BL/6 mouse model (Figure 7A). Figure 7B shows a photograph of harvested lungs from differently treated groups after the treatments. As shown in Figure 7C, the lungs of the saline-treated group showed the highest tumor nodule numbers among all groups. Due to the direct effects of killing tumor cells, the Dox- and Dox NP-treated groups showed lower tumor nodule numbers. Meanwhile, the Suc- and Suc NP-treated groups also presented lower tumor nodule numbers than the negative control, revealing that Suc could efficiently suppress the lung metastasis of melanoma. When Suc was combined with Dox, the inhibitory effects of Suc were further enhanced. The Co-NP- and Dox + Suc-treated groups both showed lower tumor nodule numbers than other separate monotherapies, indicating that Dox could enhance the inhibitory effects of Suc on the lung metastasis of melanoma. Particularly, the Co-NP-treated group showed the lowest tumor nodule numbers. In addition, the lung weight of each group presented a similar tendency of the tumor nodule numbers, which further certified that Suc could effectively suppress the lung metastasis of melanoma (Figure 7D). Furthermore, there were no significant changes in the body weight of mice in the Co-NP-treated group during the whole treatment, which further proved the favorable safety of Co-NPs in vivo. (Figure 7E).

Moreover, H&E and TUNEL staining were performed to further assess the anti-lung metastasis efficacy of Co-NPs. As shown in Figure 7F, compact nuclei of tumor cells were largely observed in the saline-treated group and reduced in the Dox-, Dox + Suc-, Dox NP-, Suc NP-, and Co-NP-treated groups. Notably, the Co-NP-treated group exhibited the largest area of intact lung tissue. Meanwhile, TUNEL staining showed similar results to the primary tumor-bearing mouse model (Figure 7G). The Co-NP-treated group presented the highest level of apoptosis, further demonstrating its strongest anti-lung metastasis efficacy.

Eventually, the immunofluorescence assay was performed to evaluate VCAM-1 expression on the lung tissues of differently treated groups. As shown in Figure 7H, the results represented a tendency almost consistently similar to the primary tumor-bearing mouse model, which further verified that Suc could efficiently inhibit VCAM-1 expression and Dox enhanced the effects of Suc.

## 4. Conclusions

In this study, dual drug-loaded nanoparticles (Co-NPs) were successfully developed using PLGA-encapsulated VCAM-1 inhibitor succinobucol and chemotherapeutic doxorubicin as hydrophobic cores and DSPE-mPEG2000 as surface modification material. The particles had a favorable size (122.4 nm), zeta potential (−6.77 mV), stability, hemocompatibility, drug release behavior, and cellular uptake efficiency. In vitro cell experiments showed that Co-NPs had potent cytotoxicity, revealing the synergistic inhibitory effects of Dox and Suc on the proliferation of B16F10 cells. Co-NPs also significantly inhibited VCAM-1 expression and migration of B16F10 cells due to Suc as a potent VCAM-1 inhibitor; this also revealed that Dox enhanced the inhibitory effects of Suc on VCAM-1 expression. Consistent with the results of in the vitro cell experiments, in vivo animal experiments also demonstrated that Co-NPs effectively suppressed not only primary melanoma but also its lung metastases, further confirming that Dox and Suc had synergistic anti-tumor and anti-metastasis efficacy in vivo. Furthermore, Co-NPs showed good in vivo safety. In summary, using PLGA nanoparticles loaded with the VCAM-1 inhibitor succinobucol and the chemotherapeutic doxorubicin provides a promising drug nano-delivery strategy combining anti-primary tumor therapy and anti-lung metastasis therapy for metastatic malignant tumors.

## Figures and Tables

**Table 1:** Critical characteristics of nanoparticles.

Nanoparticle	Size (nm)	PDI	Zeta Potential (mV)	LE % (Dox)	EE % (Dox)	LE % (Suc)	EE % (Suc)
Dox NPs	155.5 ± 5.5	0.213 ± 0.006	−7.81 ± 0.23	3.59	93.56	/	/
Suc NPs	131.6 ± 2.2	0.164 ± 0.003	−7.62 ± 0.19	/	/	4.83	98.63
Co-NPs	122.4 ± 1.1	0.245 ± 0.007	−6.77 ± 0.42	4.02	84.30	6.54	94.29

Abbreviations: PDI, polydispersity index; LE, drug loading efficiency; EE, drug encapsulation efficiency. *n* = 3.

## Data Availability

Not applicable.

## Supplementary Materials

The following supporting information can be downloaded at: https://www.mdpi.com/article/10.3390/pharmaceutics15020349/s1, Figure S1: Photograph of prepared Dox NPs, Suc NPs, and Co-NPs; Figure S2: Storage stability of nanoparticles; Figure S3: In vivo safety of nanoparticles.

## References

[1] Sung H., Ferlay J., Siegel R.L., Laversanne M., Soerjomataram I., Jemal A., Bray F. (2021). Global Cancer Statistics 2020: GLOBOCAN Estimates of Incidence and Mortality Worldwide for 36 Cancers in 185 Countries. CA Cancer J. Clin..

[2] Cohen P.A., Jhingran A., Oaknin A., Denny L. (2019). Cervical cancer. Lancet.

[3] Achrol A.S., Rennert R.C., Anders C., Soffietti R., Ahluwalia M.S., Nayak L., Peters S., Arvold N.D., Harsh G.R., Steeg P.S. (2019). Brain metastases. Nat. Rev. Dis. Prim..

[4] Kudchadkar R.R., Lowe M.C., Khan M.K., McBrien S.M. (2020). Metastatic melanoma. CA Cancer J. Clin..

[5] Valastyan S., Weinberg R.A. (2011). Tumor Metastasis: Molecular Insights and Evolving Paradigms. Cell.

[6] Dong J., Zhu C., Zhang F., Zhou Z., Sun M. (2021). “Attractive/adhesion force” dual-regulatory nanogels capable of CXCR4 antagonism and autophagy inhibition for the treatment of metastatic breast cancer. J. Control. Release.

[7] Chi J., Jiang Z., Qiao J., Peng Y., Liu W., Han B. (2019). Synthesis and anti-metastasis activities of norcantharidin-conjugated carboxymethyl chitosan as a novel drug delivery system. Carbohydr. Polym..

[8] Wang G., Sun M., Jiang Y., Zhang T., Sun W., Wang H., Yin F., Wang Z., Sang W., Xu J. (2019). Anlotinib, a novel small molecular tyrosine kinase inhibitor, suppresses growth and metastasis via dual blockade of VEGFR2 and MET in osteosarcoma. Int. J. Cancer.

[9] Kudelka A.P., Levy T., Verschraegen C., Edwards C.L., Piamsomboon S., Termrungruanglert W., Freedman R.S., Kaplan A.L., Kieback D.G., A Meyers C. (1997). A phase I study of TNP-470 administered to patients with advanced squamous cell cancer of the cervix. Clin. Cancer Res..

[10] A Shepherd F., Sridhar S.S. (2003). Angiogenesis inhibitors under study for the treatment of lung cancer. Lung Cancer.

[11] Franks M.E., Macpherson G.R., Figg W.D. (2004). Thalidomide. Lancet.

[12] Clark J.I., Moon J., Hutchins L.F., Sosman J.A., Kast W.M., Da Silva D.M., Liu P.Y., Thompson J.A., Flaherty L.E., Sondak V.K. (2009). Phase 2 trial of combination thalidomide plus temozolomide in patients with metastatic malignant melanoma: Southwest Oncology Group S0508. Cancer.

[13] Liu Y.-S., Lin H.-Y., Lai S.-W., Huang C.-Y., Huang B.-R., Chen P.-Y., Wei K.-C., Lu D.-Y. (2017). MiR-181b modulates EGFR-dependent VCAM-1 expression and monocyte adhesion in glioblastoma. Oncogene.

[14] Chen Q., Zhang X.H., Massagué J. (2011). Macrophage binding to receptor VCAM-1 transmits survival signals in breast cancer cells that invade the lungs. Cancer Cell.

[15] Chen Q., Massagué J. (2012). Molecular pathways: VCAM-1 as a potential therapeutic target in metastasis. Clin. Cancer Res..

[16] Ferjančič Š., Gil-Bernabé A.M., Hill S.A., Allen D., Richardson P., Sparey T., Savory E., McGuffog J., Muschel R.J. (2013). VCAM-1 and VAP-1 recruit myeloid cells that promote pulmonary metastasis in mice. Blood.

[17] Schlesinger M., Bendas G. (2015). Vascular cell adhesion molecule-1 (VCAM-1)-An increasing insight into its role in tumorigenicity and metastasis. Int. J. Cancer.

[18] VanHeyst K.A., Choi S.H., Kingsley D.T., Huang A.Y. (2022). Ectopic Tumor VCAM-1 Expression in Cancer Metastasis and Therapy Resistance. Cells.

[19] Colle D., Santos D.B., Hartwig J.M., Godoi M., Engel D.F., de Bem A.F., Braga A.L., Farina M. (2015). Succinobucol, a Lipid-Lowering Drug, Protects Against 3-Nitropropionic Acid-Induced Mitochondrial Dysfunction and Oxidative Stress in SH-SY5Y Cells via Upregulation of Glutathione Levels and Glutamate Cysteine Ligase Activity. Mol. Neurobiol..

[20] Meng C.Q., Somers P.K., Rachita C.L., Holt L.A., Hoong L.K., Zheng X., Simpson J.E., Hill R.R., Olliff L.K., Kunsch C. (2002). Novel phenolic antioxidants as multifunctional inhibitors of inducible VCAM-1 expression for use in atherosclerosis. Bioorganic Med. Chem. Lett..

[21] A Wasserman M., Sundell C.L., Kunsch C., Edwards D., Meng C.Q., Medford R.M. (2003). Chemistry and pharmacology of vascular protectants: A novel approach to the treatment of atherosclerosis and coronary artery disease. Am. J. Cardiol..

[22] Doggrell S.A. (2003). Experimental and clinical studies show that the probucol derivative AGI-1067 prevents vascular growth. Expert Opin. Investig. Drugs.

[23] Cao H., Zhang Z., Zhao S., He X., Yu H., Yin Q., Zhang Z., Gu W., Chen L., Li Y. (2015). Hydrophobic interaction mediating self-assembled nanoparticles of succinobucol suppress lung metastasis of breast cancer by inhibition of VCAM-1 expression. J. Control. Release.

[24] He X., Yu H., Bao X., Cao H., Yin Q., Zhang Z., Li Y. (2016). pH-Responsive Wormlike Micelles with Sequential Metastasis Targeting Inhibit Lung Metastasis of Breast Cancer. Adv. Healthc. Mater..

[25] Dan Z., Cao H., He X., Zhang Z., Zou L., Zeng L., Xu Y., Yin Q., Xu M., Zhong D. (2016). A pH-Responsive Host-guest Nanosystem Loading Succinobucol Suppresses Lung Metastasis of Breast Cancer. Theranostics.

[26] Magdy T., Jiang Z., Jouni M., Fonoudi H., Lyra-Leite D., Jung G., Romero-Tejeda M., Kuo H.-H., Fetterman K.A., Gharib M. (2021). RARG variant predictive of doxorubicin-induced cardiotoxicity identifies a cardioprotective therapy. Cell Stem Cell.

[27] Kong C.-Y., Guo Z., Song P., Zhang X., Yuan Y.-P., Teng T., Yan L., Tang Q.-Z. (2022). Underlying the Mechanisms of Doxorubicin-Induced Acute Cardiotoxicity: Oxidative Stress and Cell Death. Int. J. Biol. Sci..

[28] Bigagli E., Cinci L., D’Ambrosio M., Luceri C. (2019). Transcriptomic Characterization, Chemosensitivity and Regulatory Effects of Exosomes in Spontaneous EMT/MET Transitions of Breast Cancer Cells. Cancer Genom.—Proteom..

[29] Liu C.-L., Chen M.-J., Lin J.-C., Lin C.-H., Huang W.-C., Cheng S.-P., Chen S.-N., Chang Y.-C. (2019). Doxorubicin Promotes Migration and Invasion of Breast Cancer Cells through the Upregulation of the RhoA/MLC Pathway. J. Breast Cancer.

[30] Mohammed S., Shamseddine A.A., Newcomb B., Chavez R.S., Panzner T.D., Lee A.H., Canals D., Okeoma C.M., Clarke C.J., Hannun Y.A. (2021). Sublethal doxorubicin promotes migration and invasion of breast cancer cells: Role of Src Family non-receptor tyrosine kinases. Breast Cancer Res..

[31] Yang F., Hu Y., Shao L., Zhuang J., Huo Q., He S., Chen S., Wang J., Xie N. (2021). SIRT7 interacts with TEK (TIE2) to promote adriamycin induced metastasis in breast cancer. Cell. Oncol..

[32] Nechaeva A.M., Artyukhov A.A., Luss A.L., Shtilman M.I., Svistunova A.Y., Motyakin M.V., Levina I.I., Krivoborodov E.G., Toropygin I.Y., Chistyakov E.M. (2022). The Synthesis and Properties of a New Carrier for Paclitaxel and Doxorubicin Based on the Amphiphilic Copolymer of *N* -vinyl-2-pyrrolidone and Acrylic Acid. Macromol. Chem. Phys..

[33] Nechaeva A., Artyukhov A., Luss A., Shtilman M., Gritskova I., Shulgin A., Motyakin M., Levina I., Krivoborodov E., Toropygin I. (2022). Synthesis of Amphiphilic Copolymers of *N*-Vinyl-2-pyrrolidone and Allyl Glycidyl Ether for Co-Delivery of Doxorubicin and Paclitaxel. Polymers.

[34] Lu Z., Long Y., Li J., Ren K., Zhao W., Wang X., Xia C., Wang Y., Li M., Zhang Z. (2021). Simultaneous inhibition of breast cancer and its liver and lung metastasis by blocking inflammatory feed-forward loops. J. Control. Release.

[35] Jiang K., Song X., Yang L., Li L., Wan Z., Sun X., Gong T., Lin Q., Zhang Z. (2017). Enhanced antitumor and anti-metastasis efficacy against aggressive breast cancer with a fibronectin-targeting liposomal doxorubicin. J. Control. Release.

[36] Hua Y., Su Y., Zhang H., Liu N., Wang Z., Gao X., Gao J., Zheng A. (2021). Poly(lactic-co-glycolic acid) microsphere production based on quality by design: A review. Drug Deliv..

[37] Che J., Okeke C., Hu Z.-B., Xu J. (2015). DSPE-PEG: A Distinctive Component in Drug Delivery System. Curr. Pharm. Des..

[38] Huang J., Lai W., Wang Q., Tang Q., Hu C., Zhou M., Wang F., Xie D., Zhang Q., Liu W. (2021). Effective Triple-Negative Breast Cancer Targeted Treatment Using iRGD-Modified RBC Membrane-Camouflaged Nanoparticles. Int. J. Nanomed..

[39] Liu Y., Li Q., Xiong X., Huang Y., Zhou Z. (2018). Enhanced cellular uptake by non-endocytic pathway for tumor therapy. J. Mater. Chem. B.

[40] Zhou M., Li L., Li L., Lin X., Wang F., Li Q., Huang Y. (2018). Overcoming chemotherapy resistance via simultaneous drug-efflux circumvention and mitochondrial targeting. Acta Pharm. Sin. B.

[41] Li Q., Yang J., Chen C., Lin X., Zhou M., Zhou Z., Huang Y. (2020). A novel mitochondrial targeted hybrid peptide modified HPMA copolymers for breast cancer metastasis suppression. J. Control. Release.

[42] Merbel A.F., Horst G.V., Buijs J.T., Pluijm G.V. (2018). Protocols for Migration and Invasion Studies in Prostate Cancer. Methods Mol. Biol..

[43] Kawai H., Minamiya Y., Kitamura M., Matsuzaki I., Hashimoto M., Suzuki H., Abo S. (1997). Direct measurement of doxorubicin concentration in the intact, living single cancer cell during hyperthermia. Cancer.

[44] Sahay G., Batrakova E.V., Kabanov A.V. (2008). Different Internalization Pathways of Polymeric Micelles and Unimers and Their Effects on Vesicular Transport. Bioconjugate Chem..

[45] Behzadi S., Serpooshan V., Tao W., Hamaly M.A., Alkawareek M.Y., Dreaden E.C., Brown D., Alkilany A.M., Farokhzad O.C., Mahmoudi M. (2017). Cellular uptake of nanoparticles: Journey inside the cell. Chem. Soc. Rev..

[46] Kamaly N., Yameen B., Wu J., Farokhzad O.C. (2016). Degradable Controlled-Release Polymers and Polymeric Nanoparticles: Mechanisms of Controlling Drug Release. Chem. Rev..

[47] Jabir N.R., Tabrez S., Ashraf G.M., Shakil S., Damanhouri G.A., Kamal M.A. (2012). Nanotechnology-based approaches in anticancer research. Int. J. Nanomed..

[48] Hynes R.O. (2011). Metastatic Cells Will Take Any Help They Can Get. Cancer Cell.

[49] Zhang X., Liu C., Hu F., Zhang Y., Wang J., Gao Y., Jiang Y., Zhang Y., Lan X. (2018). PET Imaging of VCAM-1 Expression and Monitoring Therapy Response in Tumor with a ^68^Ga-Labeled Single Chain Variable Fragment. Mol. Pharm..

