# Supplementary material for: PLGA Nanoparticles Containing VCAM-1 Inhibitor Succinobucol and Chemotherapeutic Doxorubicin as Therapy against Primary Tumors and Their Lung Metastases
Source: Pharmaceutics. 2023 Jan 20;15(2):349. doi: 10.3390/pharmaceutics15020349 (PMC9958791; doi:10.3390/pharmaceutics15020349)
Supplement: Supplementary file 1 [file pharmaceutics-15-00349-s001.zip › pharmaceutics-2091295-supplementary.pdf]

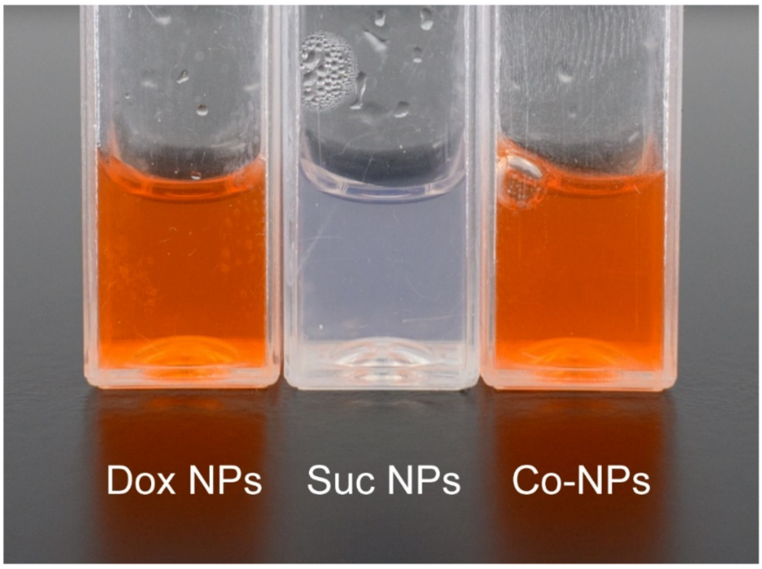

Figure S1. Photograph of prepared Dox NPs, Suc NPs, and Co-NPs.

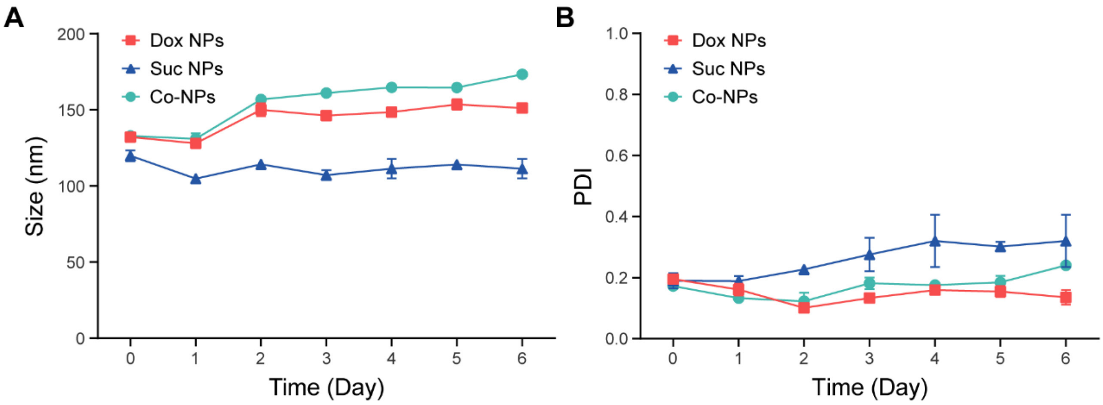

Figure S2. Storage stability of nanoparticles. **A.** Size of three kinds of NPs in PBS (pH 7.4) at 4 °C in 7 days. **B.** PDI of three kinds of NPs in PBS (pH 7.4) at 4 °C in 7 days.

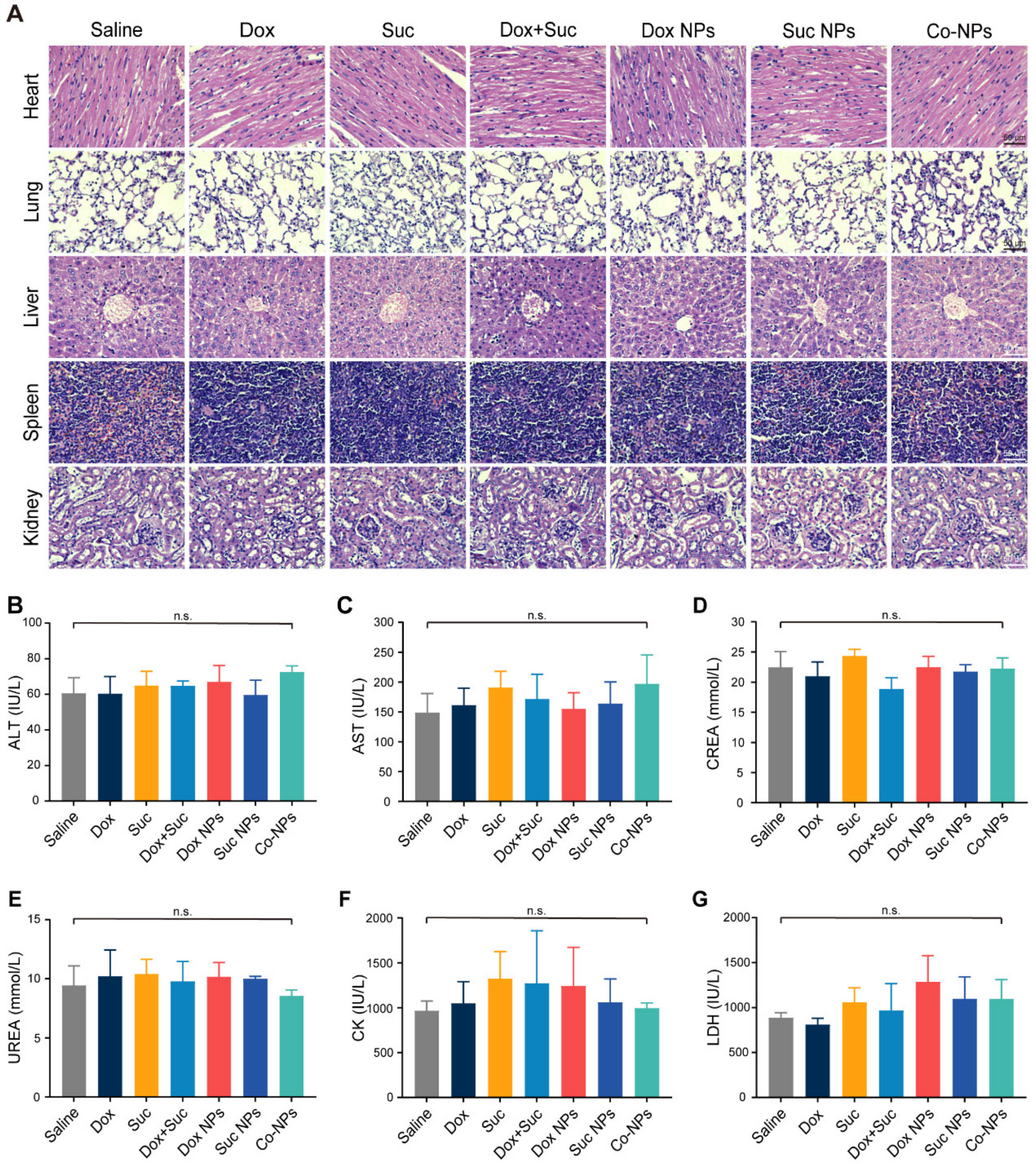

Figure S3. *In vivo* safety of nanoparticles. **A.** H&E staining of the harvested heart, lung, liver, spleen and kidney after different treatments. Scale bar = 50 μm. **B-G.** Hematological analysis of ALT (**B**), AST (**C**), CREA (**D**), UREA (**E**), CK (**F**) and LDH (**G**) in C57BL/6 mice after different treatments. Each value represents the mean ± SD (n = 3). (n.s., not significant)
